# Effect of Publicly Reporting Performance Data of Medicine Use on Injection Use: A Quasi-Experimental Study

**DOI:** 10.1371/journal.pone.0109594

**Published:** 2014-10-14

**Authors:** Xuan Wang, Yuqing Tang, Xiaopeng Zhang, Xi Yin, Xin Du, Xinping Zhang

**Affiliations:** School of Medicine and Health Management, Tongji Medical College, HuaZhong University of Science and Technology, Wuhan, HuBei Province, China; Canadian Agency for Drugs and Technologies in Health, Canada

## Abstract

**Background:**

Inappropriate use of prescribing pharmaceuticals, particularly injections, not only affects the quality of medical care, but also leads to an increase in medical expenses. Publicly reporting performance data of medical care is becoming a common health policy tool adopted to supervise medical quality. To our knowledge, few studies about public reporting applied to medicine use have been reported. This study intended to introduce public reporting in the field of medicine use, and evaluate the effect of publicly reporting performance data of medicine use on the use of injections.

**Methods:**

The research sites were 20 primary healthcare institutions in Q City, Hubei. By matching, the institutions were divided into the intervention group and control group. A quasi-experimental design was applied in this study. In the intervention group, the performance data of medicine use were publicly reported. The injection prescribing rates of the two groups before and after intervention were measured and compared. Difference-in-difference method and logistic regression were employed to estimate the effect of public reporting on injection use.

**Results:**

Public reporting led to a reduction of approximately 4% in the injection prescribing rate four months after intervention (OR = 0.96; 95%CI: 0.94, 0.97). The intervention effect was inconsistent in each month after intervention, and it was most positive in the second month after intervention (OR = 0.90; 95%CI: 0.89, 0.92).

**Conclusions:**

In general, publicly reporting performance data of medicine use may have positive effects on injection use to some extent. Further research is needed to investigate the mechanism by which public reporting influences injection use. Comprehensive measures are also necessary to promote the rational use of injections.

## Introduction

Rational use of medicines refers to the appropriate use of medicines. Rational use requires that patients receive the appropriate medicine, in the proper dose, for an adequate period of time, and at the lowest cost to them and their community. However, the World Health Organization (WHO) estimates that more than half of all medicines are prescribed, dispensed, or sold inappropriately [1]. The consequences of the irrational use of medicines include adverse drug reactions, drug resistance, protracted illness and even death [2], [3]. The financial burden arising from the irrational use of medicines is profound and often unexpectedly high [4]. Inappropriate use of prescribing pharmaceuticals occurs commonly in healthcare institutions worldwide, especially in developing countries [5]. Overuse of injections is a common form of the inappropriate use of prescribing pharmaceuticals. According to prior research, overuse of injections occurs commonly in healthcare institutions in China, especially in primary healthcare institutions [6]–[9]. The unnecessary use of injections not only wastes medical resources but also increases the patient's risk of infection by viruses, such as hepatitis C and AIDS [10]. Effective measures are necessary to improve injection use.

WHO has proposed 12 core policies to promote the rational use of medicines. The seventh item is supervision, audit, and feedback [11]. Audit and feedback is widely used as a strategy to improve medical quality. It is based on the belief that knowledge of poor performance either by administrators or physicians themselves will result in behavioral change that improve performance [12], [13]. Evidence suggested that the audit and feedback of antibiotic prescribing can help reduce the unnecessary antibiotic prescribing [14], [15]. In addition, publicly reporting performance results has greater effects on quality improvement than performance evaluation alone [16]. Public reporting of performance data is becoming a common health policy tool to supervise medical quality [17]. The implementation of public reporting systems started in New York in the mid-1980s [18]. Many other Western countries then started to implement public reporting to their healthcare systems [19], [20]. To improve the transparency of medical health services, the Ministry of Health of China issued a bulletin named *Management measure about information disclosure of medical health service institution* in 2010. Studies suggested that publicly reporting performance data can stimulate quality improvement activity at the hospital level [21], [22]. The public reporting of performance data has been proposed as a mechanism for improving quality of care by providing more transparency and greater accountability of healthcare providers [23].

According to Berwick and colleagues' framework for quality improvement, public reporting can improve performance through two pathways (the selection pathway and change pathway). In the selection pathway, patients compare the publicly reported performance data, and select the providers with better performance. The selection pathway is interconnected with the change pathway by a provider's motivation to protect or enhance market share. In the change pathway, providers identify areas in which they underperform and improve their performance [24], [25]. Simultaneously, public reporting may change the interpersonal factors within the context of patient care. A study in Los Angeles community clinics indicated that displaying poster-sized commitment letters in examination rooms decreases inappropriate antibiotic prescribing [13].

Publicly reporting performance data of medicine use is a way of supervision that connects public reporting with the audit and feedback of prescribing. According to prior research, overuse of injections most commonly occurs in primary healthcare institutions in China [6]–[9]. Consequently, we chose primary healthcare institutions as our research sites. We employed a quasi-experimental study to estimate the effect of publicly reporting performance data of medicine use on the use of injections. The rationale for publicly reporting performance data relies largely on the belief that the public reporting performance data will lead to behavioral change and improve quality [26]. Therefore, we generated a hypothesis that the overuse of injections would be reduced by publicly reporting performance data of medicine use.

## Methods

### Ethics Statement

This study and its consent procedure were approved by the Ethics Committee of Tongji Medical College, Huazhong University of Science and Technology. Patient information was anonymized and de-identified prior to analysis.

### Research Design

Hubei Province is located in south central China, which has a population of 59.88 million. Q City is located in central Hubei Province, and it is a typical city of this province. All the 20 primary healthcare institutions in Q City were identified as participating organizations. The Technique for Order Preference by Similarity to an Ideal Solution (TOPSIS) was employed to match the 20 institutions. The TOPSIS score was generated according to nine indicators, including the service population, number of approval beds, and number of physicians. Two institutions with the closest score were paired, and randomly located into the control group and intervention group. Twenty institutions were matched into ten pairs, with 10 institutions into the intervention group and 10 institutions into the control group. In the intervention group, the performance data of medicine use were publicly reported. This information was not disclosed in the control group. A quasi-experimental design was applied in this study. We collected all the electronic prescriptions, four months before and after intervention, from the electronic information system. The injection prescribing rates of the two groups before and after intervention were measured and compared. Difference-in-difference method and logistic regression were employed to estimate the effect of public reporting on injection use.

### Intervention Measures

The time span of the intervention was from 1 November 2013 to 31 February 2014. In the intervention group, the injection prescribing rate and ranking were publicly reported at the level of individual physicians and institutions according to the uniform standard. The injection prescribing rate is used to estimate whether the use of injection is rational, and defined as the number of prescriptions using injections divided by the total number of prescriptions [27]. Based on the premise that the actual injection prescribing rate is higher than the standard suggested by WHO, a lower injection prescribing rate indicates better performance, whereas a higher injection prescribing rate indicates poor performance. The physician or institution with better performance ranks first, and the physician or institution with poor performance ranks behind. The information was publicly reported on bulletin boards and in brochures. The bulletin boards were placed in the outpatient service hall so that physicians or patients could easily see the public information when entering the outpatient service hall. The brochures for patients were placed on the service counter in the outpatient service hall. The public information was updated monthly. At the beginning of each month, we publicly reported the performance data of the last month. For example, in February 2014, the performance data in January 2014 was publicly reported for a whole month. On the bulletin board and in the brochure, we used two forms to display the information. One form was for the individual physician, and the other was for the institution. The content of forms consisted of the name of the physician or institution, injection prescribing rate, and ranking. Below the forms, some knowledge on rational medicine use was introduced.

This study was conducted in collaboration with the local health bureau. The local health bureau provided the database of electronic prescriptions. Our research team was responsible for data collection and calculation, making bulletins and brochures, and publicly reporting the information. Before the start of public reporting, the local health bureau informed the institution leader about the implementation of public reporting by meeting and issuing documents. The institution leader then informed the physicians by meeting. Our research team delivered the public performance results to the institution leaders and the physicians by notification every month. In this study, no financial incentive measure, such as giving financial rewards or penalties to the physician or institution with better performance or poor performance, was adopted.

### Outcome Measure

To compare the changes in injection use between the two groups over time, the injection prescribing rates of the two groups were measured before and after intervention (from 1 July 2013 to 31 February 2014).

### Data Collection

From the database of electronic prescriptions of the local health bureau, we obtained all the electronic prescriptions of the two groups four months before and after intervention (from 1 July 2013 to 31 February 2014). We collected 1,566,661 effective electronic prescriptions, from which we used the information, including patient age, patient gender, and whether or not to use injection, for statistical analysis.

### Statistical Analysis

Difference-in-difference method is usually used to evaluate the net effect of a policy. The research subjects were divided into the intervention group and control group. The variations in an index of the two groups before and after intervention were calculated. The difference between these two variations (the so-called difference in difference) reflects the net effect of the intervention policy (Table 1). In this study, the index was the injection prescribing rate.

**Table 1:** Design of the difference-in-difference method.

Group	Before intervention	After intervention	D	DID
**Intervention group**	A1	A2	ΔA = A2−A1	ΔA−ΔB
**Control group**	B1	B2	ΔB = B2−B1	

We considered all the effective prescriptions in the research period as observation units. Logistic regression equation was established as follows: (the variables involved in the equation and their definitions are shown in Table 2)

**Table 2:** Definition of independent variables and dependent variable.

Variable	Definition
**Dependent variable (** ***Y*** **)**	
**whether injection is used in a prescription**	0 not use, 1 use
**Independent variables (** ***X*** **)**	
**after**	0 before intervention, 1 after intervention
**group**	0 control group, 1 intervention group
**After × group**	multiply after by group
**gender**	0 female, 1 male
**age**	year as a unit*

*For example, if the patient is six months old, the age should be converted to 0.5.

logit(*P*)  = *β*_0_+*β*_1_×after+*β*_2_×group+*β*_3_×after×group+*β*_4_×*x*_i_

where *P* is the probability of “*Y*  =  1” (injection is used in a prescription), *β*_1_ is the change before and after intervention, *β*_2_ is the difference between the two groups, *β_3_* is the net effect of intervention, and *x*_i_ is a set of explanatory variables, including patient gender and patient age. Statistical analysis was conducted using STATA (version 12.0).

## Results

### Data Description and Distribution

The total number of effective electronic prescriptions was 1,566,661. Among them, 813,478 were from the control group and 753,183 were from the intervention group. Male patients accounted for 53.97% in the control group and 55.18% in the intervention group. Chi-square test showed that the difference between the two groups was statistically significant (P<0.05). In the control group, the average age of patients was 34.11 years, whereas that in the intervention group was 30.60 years. According to the *t*-test of two independent samples, the difference between the two groups was statistically significant (P<0.05). The distribution of data is shown in Table 3.

**Table 3:** Basic characteristics of the sample.

Variables	Control group		Intervention group	
	BI	AI	BI	AI
**Number of prescriptions**	397,722	415,756	372,540	380,643
**Gender (%)**				
**Male**	54.11	53.84	55.80	54.59
**Age (mean)**	34.68	33.57	30.42	30.77

Note: BI means before intervention, and AI means after intervention.

### Results of Descriptive Analysis on Injection Use

The data of four months before intervention were used as the baseline. We compared the data of four months after intervention with the baseline data. The injection prescribing rate in the control group was higher than that in the intervention group at baseline. It decreased from 73.40% to 69.32% in the control group, whereas decreased from 66.77% to 60.87% in the intervention group. Compared with the control group, the reduction was more obvious in the intervention group.

**Figure 1:**
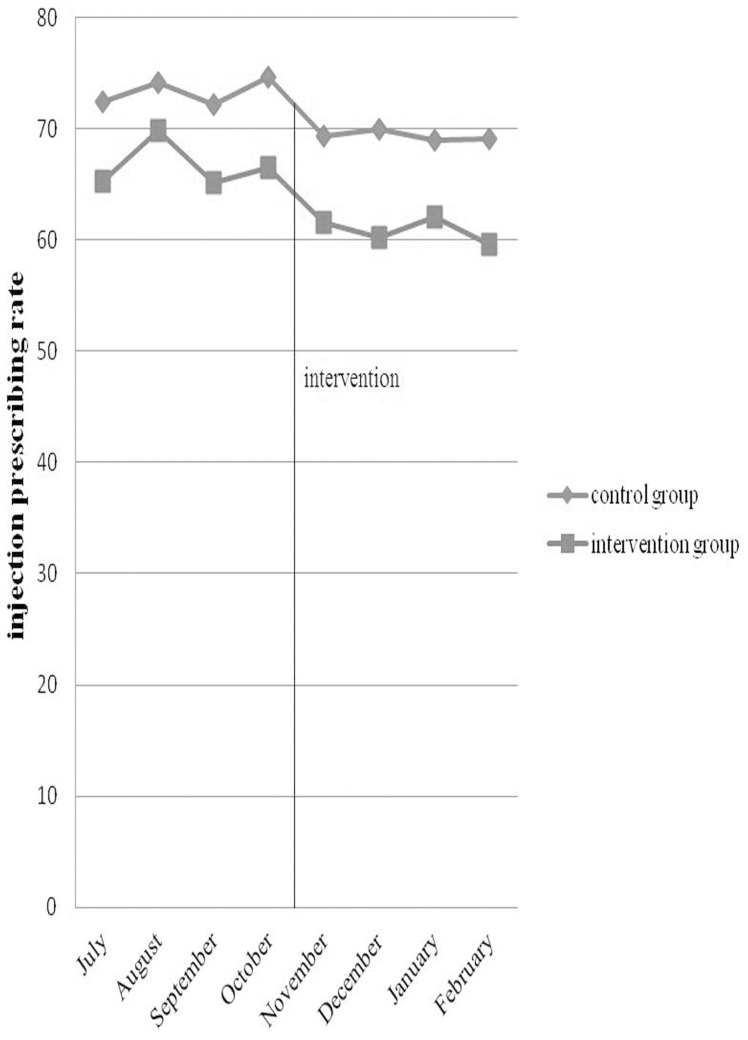
Change in injection prescribing rate by month.

As shown in Figure 1, the injection prescribing rate varied over time. Compared with the control group, the injection prescribing rate declined most obviously in the second month after intervention in the intervention group.

### Results of Logistic Regression on Injection Use

The data of four months before intervention were used as the baseline. We compared the data of four months after intervention with the baseline data. Difference-in-difference method and logistic regression were employed. The variables in the equation and their definitions are shown in Table 2, and the results of our analysis are shown in Table 4. When the effects of patient gender and patient age were controlled, the intervention led to a reduction of approximately 4% in the injection prescribing rate (OR = 0.96; 95%CI: 0.94, 0.97). In terms of injection prescribing rate, male patients were slightly lower than female patients, and older patients were lower than younger patients.

**Table 4:** Logistic regression results of injection use.

Variables	OR	95%CI	P value
**After × group**	0.96	0.94–0.97	0.00
**after**	0.81	0.80–0.82	0.00
**group**	0.70	0.69–0.71	0.00
**gender**	0.92	0.91–0.92	0.00
**age**	0.99	0.99–0.99	0.00

To compare the intervention effect of each month after intervention, the data of four months before intervention were used as the baseline, whereas the data of the first month, the second month, the third month, and the fourth month after intervention were compared with the baseline data respectively. Difference-in-difference method and logistic regression were employed. The variables in the equation and their definitions are the same as above (Table 2), and the results are shown in Table 5. The value of OR is for the variable after × group, which reflects the net effect of intervention.

**Table 5:** Comparison of intervention effect in each month after intervention.

Time	OR	95%CI	P	Sample size
**First month**	0.98	0.96–1.00	0.07	951,785
**Second month**	0.90*	0.89–0.92	0.00	976,860
**Third month**	1.02*	1.00–1.05	0.02	989,944
**Fourth month**	0.92*	0.90–0.94	0.00	958,858

Note: The value of OR is for the variable after × group.

*P<0.05.

The intervention effect was inconsistent in each month after intervention, and it was most positive in the second month after intervention (OR = 0.90; 95%CI: 0.89, 0.92).

## Discussion

This study provided reliable evidence of the effect of publicly reporting performance data of medicine use on injection use in primary healthcare institutions in Q City. Prescribing practices are influenced by several factors [28]. To distinguish the intervention effect from the confounding factors, we chose a quasi-experimental design as the research method, and established a control group. According to the principle of quasi-experimental design, the control group also needs to be selected based on the comparable principle [29]. To ensure a similar distribution of institutional characteristics between the two groups, TOPSIS method was used to match the two groups. If the two groups are close in geographical position in a quasi-experiment, the control group is easily contaminated by the intervention. That is to say, the control group will be influenced to implement similar measures, so the intervention effect is underestimated [29]. Our research team regularly inspected the two groups during the study period. On the one hand, we supervised the intervention group to implement the intervention measures according to the requirements. On the other hand, we avoided the control group by implementing similar measures through effective supervision. In the quasi-experimental design, difference-in-difference method is an analytical method of high internal validity [30]. Thus, we employed difference-in-difference method for statistical analysis. According to previous research [31], patient gender and patient age can affect injection use. We used these two factors as independent variables in the logistic regression equation, and eliminated their influence on the intervention effect. Therefore, our research results are reliable. In our collaboration with the local health bureau, we implemented a quasi-experimental design and contributed to the small but growing body of evidence about the effect of public reporting on injection use.

In general, the intervention showed a moderate effect in reducing the overuse of injections. When we compared the intervention effect of each month after the intervention, we observed that it was inconsistent, and it was most positive in the second month after intervention (OR = 0.90; 95%CI: 0.89, 0.92). Given that the overuse of injection is related to injection-associated infections, a reduction in the use of injections can also reduce the risk of spreading blood-borne viruses and infusion site infections [32]. However, we did not explore the direct mechanisms by which public reporting can influenced injection use. According to the framework of “two pathways” for quality improvement, public reporting can improve performance through two pathways (the selection pathway and change pathway). In selection pathway, patients compare the publicly reported performance data, and select the providers with better performance. In our study, patients can understand the performance data from bulletin boards and brochures. If patients can recognize the information and select the physicians with better performance, the physicians will be pressured to reduce the use of injections in order to protect or enhance market share. However, the selection pathway works well on the condition that patients can understand the information and identify that the information is important to them [24]. For example, patients who prefer injections will not regard a high injection prescribing rate as poor performance. We attached some knowledge about the rational use of medicines in the public information to strengthen patients' understanding. However, given the lower educational level of patients in the study sites, the selection pathway may play a limited role. In the change pathway, the physicians can identify areas in which they underperform. They will alter their behavior to improve their performance for self-image, peer pressure, and public reputation [16], [33]. However, the acceptability of public performance data by physicians may be an important factor influencing the motive of behavioral change [24]. In our study, the performance data were publicly reported by our research team. Relatively speaking, physicians in China may be more likely to accept the information publicly reported by the institute leader. In addition, the change pathway works better when the institute leader uses the public information, because the institute leader has the authority to employ relevant measures to improve performance [34]. The relevant measures generally include leadership to inspire performance improvement, education and training, and organizational incentives [24]. In our study, the physicians were praised or criticized by the institution leader according to their good or poor performance, which may play some role in promoting physicians' behavioral change. Research demonstrated that public reporting and pay for performance have modestly greater influences on quality improvement than public reporting alone [35]. The intervention effect may be more positive if some financial incentive measures were adopted in our study. According to the prior research, audit and feedback coupled with professional education can significantly reduce off-guideline antibiotic use [14]. In our study, we merely attached some knowledge about the rational use of medicines on bulletin boards and in brochures. Comparatively speaking, professional education may have a greater effect on behavioral change. Research demonstrated that successful public reporting requires the design and implementation of a reporting system appropriate for its purpose [21]. In our study, the public content and intervention purpose were consistent. However, both the accessibility and effectiveness of the intervention style need further investigation. A study in Los Angeles community clinics indicated that displaying poster-sized commitment letters in examination rooms can decrease inappropriate antibiotic prescribing [13]. Our study differed from this Los Angeles study in terms of the public content and public locations. Compared with poster-sized commitment letters, the performance data in our study were more directly related to the physicians' reputation. The public location was the outpatient service hall in our study, whereas it was the examination rooms in the Los Angeles study. Compared with the information in the outpatient service hall, that in the examination room may deliver a stronger message to physicians. These studies suggested that the content and style of public reporting, as well as corresponding incentive measures, influence the implementation effect of public reporting. The mechanism by which public reporting exerts its function on behavioral changes has yet to be studied.

In this study, we also observed that the level of the injection prescribing rate after intervention was still much higher than the standard suggested by WHO for developing countries (13.4% to 24.1%). First and foremost, the financial incentive for injection use may be the most important reason for injection overuse. In the 20 primary healthcare institutions, fee-for-service is the main payment method. Under this payment system, the “information gap” between physicians and their patients allows physicians to induce demand for their services [36]. In addition, healthcare providers who administer injections are paid higher fees than what they would be paid to dispense medicines [37], thereby leading to the unnecessary use of injections. Although performance data of medicine use were shared by patients through public reporting, the effect on narrowing the “information gap” was limited. The payment mechanism has significant effects on clinical decision-making [38], and government subsidies may have positive effects on injection use [9]. Thus, China must reform its healthcare financing and payment system to remove the profit incentives in prescriptions [37]. In addition, the phenomenon of patient demand-driven use of injections occurs commonly in primary healthcare institutions in research sites. In many cases, patients prefer injections because they believe them to be stronger and faster medications; they also believe that physicians regard injections to be the best treatment. Consequently, physicians overprescribe injections because they believe that this practice best satisfies patients [39]. This belief may be another important reason for the overuse of injections. The “interactional group discussions” conducted in Indonesia and India suggested that better communication between patients and physicians can result in physicians to prescribe fewer injections [40], [41]. Therefore, communication between patients and physicians should be strengthened to reduce the overuse of injections.

Our study has some limitations: We evaluated the intervention effect four months after intervention. Considering that time is needed for the intervention effects to manifest completely, the intervention effect may be underestimated. We did not analyze the disease spectrum of the two groups, which would influence the reliability of the research results. However, the 20 institutions from the same city were matched by TOPSIS method and randomly allocated to the intervention group and control group, which allowed us to presume that the disease spectrum of the two groups was relatively balanced in a relatively short time period. The study sites were primary healthcare institutions; therefore, the conclusions drawn from this research must be carefully generalized to other types of healthcare institutions.

This paper provides a quasi-experimental evaluation of the effect of publicly reporting performance data of medicine use on injection use. In general, publicly reporting performance data of medicine use may have positive effects on injection use to some extent. Further research is needed to investigate the mechanism by which public reporting can influence injection use. Comprehensive measures are also necessary to promote the rational use of injections.
